# A rare presentation of aspergillus infection as empyema thoracis

**DOI:** 10.4103/0970-2113.59265

**Published:** 2010

**Authors:** Manoj K Goel, Deven Juneja, Satinder K Jain, Saikiran Chaudhuri, Ajay Kumar

**Affiliations:** *Department of Pulmonology, Critical Care and Sleep Medicine, Delhi Heart and Lung Institute 3 MM - II Panchkuian Road, New Delhi - 110 055, India*

**Keywords:** Aspergillosis, aspergillus empyema, multiloculated empyema

## Abstract

A 57-year-old diabetic and hypertensive man presented with a short history of fever, dry cough and right side chest pain. A chest radiograph showed right pleural based homogenous shadow in middle and lower zones with obliteration of right costo-phrenic angle suggestive of right side effusion. Aspiration of pleural fluid revealed frank pus for which inter-costal tube drainage was performed. Due to persistence of empyema, the patient was subjected to thoracoscopy. Thoracoscopy showed multiloculated empyema. Thoracoscopic pleural biopsy and fluid showed septate fungal hyphae. Thoracotomy and parietal pleurectomy, with resection of part of right lower lobe, was carried out. Pleural fluid, pleural and lung tissue culture grew *Aspergillus fumigatus*. The patient showed good recovery with voriconazole after thoracotomy.

## INTRODUCTION

*Aspergillus* species is one of the commonest causes of fungal infections.[1] Three distinctive patterns of aspergillus-related lung diseases are recognized; saprophytic infestation of airways, cavities and necrotic tissue e.g. aspergillomas; allergic manifestations such as extrinsic allergic alveolitis, asthma, allergic bronchopulmonary aspergillosis (ABPA), bronchocentric granulomatosis and chronic eosinophilic pneumonia; and airway and tissue invasive disease called invasive aspergillosis.[2] The aspergillus empyema is a rare clinical entity[3] and not included in classification of aspergillus related lung diseases. Empyemas are rare presentations of fungal infection. We report a case of aspergillus empyema thoracis in a previously healthy male.

## CASE REPORT

A 57-year-old male presented with complaints of low grade fever for 10 days; dry cough, progressive breathlessness and right-sided chest pain of seven days duration. There was no history of wheezing or hemoptysis. There was no past history of any cardiac illness, pulmonary tuberculosis, COPD, blood transfusion or previous hospitalization. The patient gave history of diabetes mellitus diagnosed 10 years back and presently well-controlled on oral hypoglycemic agents. He was also a known hypertensive for seven years, well-controlled with drugs.

On physical examination, the patient was conscious and alert with a pulse rate of 110 beats per minute, blood pressure of 140/70 mmHg, and a respiratory rate of 36 breaths per minute. The accessory respiratory muscles were active. The patient was febrile with temperature 100.8°F. There was no pallor, clubbing, lymphadenopathy, icterus, cyanosis, or pedal edema. Examination of the chest revealed reduced movements on right side, and stony dull note in right infrascapular, infra-mammary and axillary regions with decreased breath sounds. Rest of the physical examination was unremarkable.

**Figure 1:**
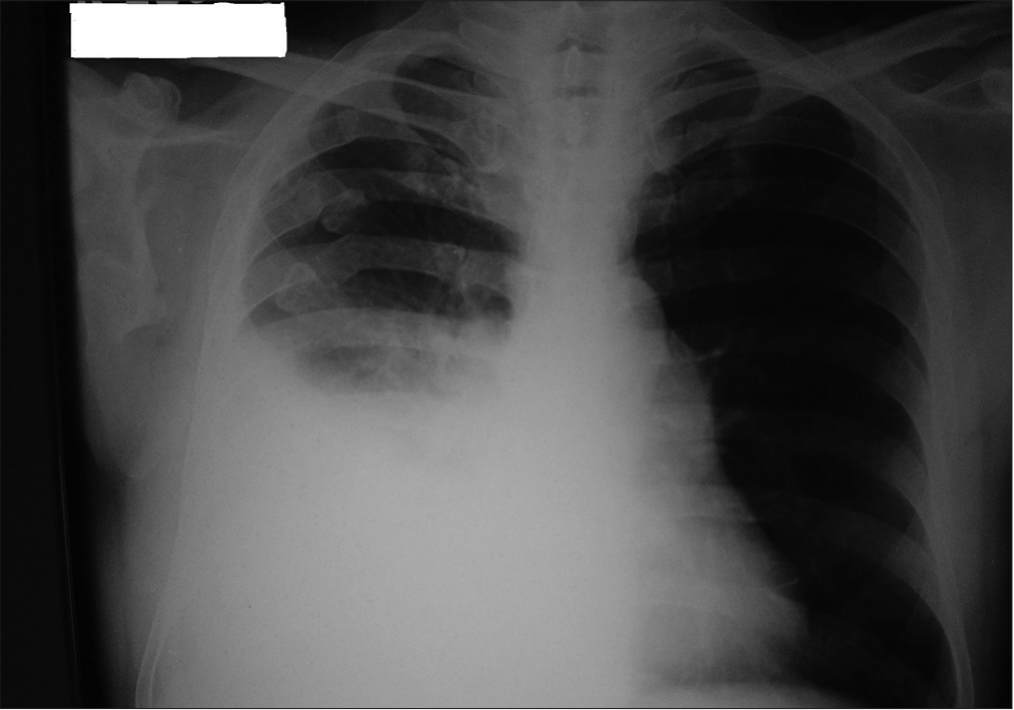
Chest X-ray on admission shows right pleural based homogenous shadow in middle and lower zones with obliteration of right costo-phrenic angle

Investigations showed hemoglobin-10.9 gm%, total leukocyte count-11,400/cu mm, differential leukocyte count-P_78_ L_06_ M_5_ E_11_, erythrocyte sedimentation rate -38 mm, random blood sugar-505 mg%, blood urea-89 mg%, serum creatinine-2.3 mg%, serum proteins-5.4 gm%, albumin-2.8 gm%, alanine aminotransferase-128 IU/l, aspartate aminotransferase-142 IU/l. Urine routine examination showed albumin in traces and sugar-4+ and ketones were absent. Electrocardiograph showed sinus tachycardia. Arterial blood gases were suggestive of uncompensated metabolic acidosis (pH: 7.297, partial pressure of carbon dioxide: 23 mmHg, partial pressure of oxygen: 119 mmHg, bicarbonate: 10.9 mEq/L) which might have been due to severe sepsis. His chest radiograph [Figure 1] showed right pleural effusion. A diagnostic thoracocentesis was performed and thin pus was aspirated. Pleural fluid cell count was 4200 (P-73%, L-18%, M-09%), glucose: 134 mg%, protein: 5.2 gm%, and lactate dehydrogenase: 1621 IU/l. Acid fast bacilli (AFB) smear and Gram's stain were negative, and pyogenic culture was sterile.

**Figure 2:**
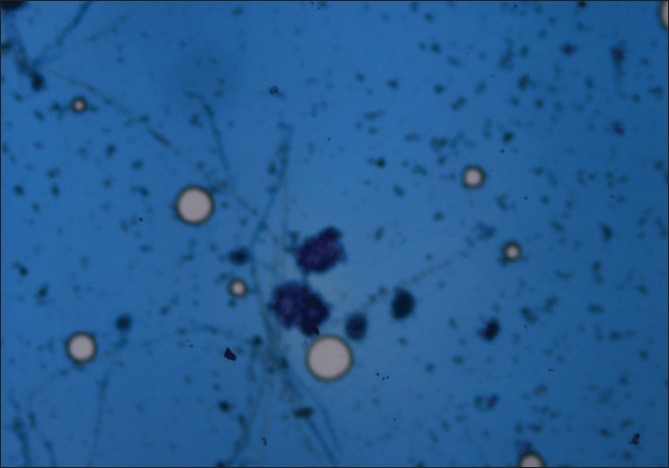
Pleural fluid smear–lactophenol blue wet mount shows fungal hyphae with aspergillus heads

**Figure 3:**
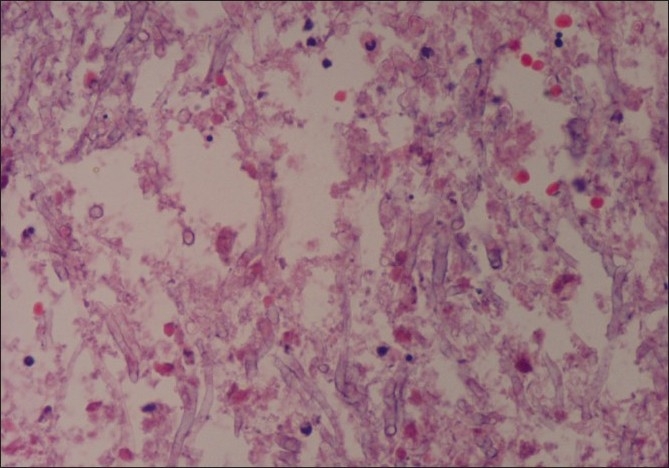
Pleural biopsy H and E (10×), stain–aspergillus hyphae

Early initiation of a combination of empirical intravenous antibiotics, amoxycillin-clavulanic acid and cefepime, was started. Inter-costal chest tube drainage was done and about 1500 ml thin pus drained. Even after four days of treatment there was no clinical improvement as fever persisted and patient remained tachypnoeic, toxic, febrile and breathless. Inter-costal drainage of about 150-200 ml of thin pus was noted daily. Chest ultrasonography (USG) was suggestive of right multi-loculated empyema. Contrast enhanced computed tomography (CT) scan of chest showed right sided empyema with multiple loculations but no pleural thickening. There was no mediastinal lymphadenopathy or lung parenchymal involvement. Mantoux test was negative. Medical thoracoscopy was performed and another 750 ml of thin pus aspirated. A large number of fibrinous adhesions with multiple loculations were seen. Diaphragm and pleural surfaces were lined with fibrinous exudates. Pleural fluid and pleural biopsy specimens were sent for pyogenic, fungal and AFB culture. Pleural fluid smears[Figure 2] showed septate fungal hyphae. Intravenous voriconazole was started with a loading dose of 400 mg twice on first day followed by 200 mg twice daily. He was taken up for surgical intervention due to persistent toxemia and multiloculated empyema. Thoracotomy revealed tough fibrinous adhesions with multiloculated fluid collection with pleural thickening. The right lower lobe showed multiple superficial cavitatory lesions measuring one-two cms. Complete parietal pleurectomy was done along with surgical resection of part of the right lower lobe. Pleural fluid, pleural and lung tissue cultures grew *Aspergillus fumigatus*, whereas the bacterial cultures and AFB smears were negative in all the samples. The hematoxyllin and eosin staining of pleural biopsy sample was positive for aspergillar hyphae [Figure 3]. The postoperative period was uneventful and he was continued with voriconazole. His general condition improved rapidly. The toxemic state, fever and breathlessness subsided. His kidney function tests settled to normal. The patient was discharged after 14 days on oral Voriconazole which was continued for three months. He was followed up to eight months and showed good clinical and radiological recovery.

## DISCUSSION

Aspergillosis is a large spectrum of diseases caused by members of the *Aspergillus* genus. *Aspergillus fumigatus* is the most commonly isolated species, followed by *Aspergillus flavus* and *Aspergillus niger*. The clinical manifestation and severity of the disease depends upon the immunologic state of the patient. Aspergillus empyema thoracis is uncommon and caused by rupture of an aspergilloma cavity or as a complication of a preexisting chronic empyema.[4] A number of factors which predispose patients to fungal infection include diabetes, glucocorticoid therapy, chemotherapy, acquired immune deficiency syndrome, hematologic malignancies,[5] previous hospitalizations especially in ICUs, pre-existing pulmonary tuberculosis, bronchopleumal fistula, pleural intubation or drainage, and lung resection.[6] Many patients have a previous history of multiple antibiotic therapy administration. In our case, the infection was community acquired with no history of previous hospitalization. The patient was apparently healthy and had only one predisposing factor, long standing diabetes, which was also adequately controlled.

The patients with pleural aspergillosis generally have a prolonged history, and the diagnosis may be suggested by growth of *Aspergillus* species from clinical specimens; failure to detect other bacterial, fungal, or mycobacterial pathogens and failure to respond to antibacterial or anti-mycobacterial therapy. There are no characteristic radiographic appearances described for the diagnosis of pleural aspergillosis. The radiological abnormalities include pleural effusion with upper lobe infiltrates, cavities and mycetomas. The patient, in this case, was earlier quite healthy and had a shorter history of only 10 days duration. There was no evidence of lung parenchymal involvement on chest X-ray as well as CT scan. A couple of small cavities could be detected only when thoracotomy was performed. The diagnosis could be confirmed because the pleural fluid and lung tissue cultures grew *Aspergillus fumigatus*.

Anti-fungal drugs like miconazole, nystatin and amphotericin B have been used both parenterally and by intra-pleural infusion, but treatment over several months is often required to sterilize the pleura.[6] Some newer anti-fungals like posaconazole[7] and voriconazole[8] are also effective. But, the success rates continue to be low, only 20-27%,[6–9] because of poor pre-morbid state and delayed diagnosis. The surgical removal of aspergillosis-infected pleura and adjacent pulmonary disease provides an effective cure.[6] This patient was critically ill, but had good recovery with voriconazole possibly mainly because of early diagnosis, prompt surgical intervention and his relatively good pre-morbid physical state.

In conclusion, Aspergillus empyema thoracis is uncommon and caused by rupture of an aspergilloma cavity or as a complication of a pre-existing chronic empyema. Empyemas of fungal origin are associated with high mortality rates but early administration of anti-fungal agents and pleural drainage might be helpful in improving the outcome. Response to anti-fungal agents is slow and may be incomplete and only aggressive resection can provide effective long term palliation. Hence, a high index of suspicion is required to ensure timely diagnosis and treatment of this potentially lethal condition.
